# The prevalence of interstitial lung disease in rheumatoid arthritis: a systematic review

**DOI:** 10.1093/rap/rkag045

**Published:** 2026-04-11

**Authors:** Marie Vermant, Thomas Pauwels, Tine Follet, Laurens J De Sadeleer, Nico De Crem, Barbara Neerinckx, Alexandros Kalkanis, Wim A Wuyts, Patrick Verschueren

**Affiliations:** Department of Chronic Diseases and Metabolism, KU Leuven, Leuven, Belgium; Pulmonology Department, University Hospitals Leuven, Leuven, Belgium; Rheumatology Department, University Hospitals Leuven, Leuven, Belgium; Department of Chronic Diseases and Metabolism, KU Leuven, Leuven, Belgium; Pulmonology Department, University Hospitals Leuven, Leuven, Belgium; Department of Chronic Diseases and Metabolism, KU Leuven, Leuven, Belgium; Pulmonology Department, University Hospitals Leuven, Leuven, Belgium; Pulmonology Department, University Hospitals Leuven, Leuven, Belgium; Rheumatology Department, University Hospitals Leuven, Leuven, Belgium; Department of Development and Regeneration, KU Leuven, Leuven, Belgium; Department of Chronic Diseases and Metabolism, KU Leuven, Leuven, Belgium; Pulmonology Department, University Hospitals Leuven, Leuven, Belgium; Department of Chronic Diseases and Metabolism, KU Leuven, Leuven, Belgium; Pulmonology Department, University Hospitals Leuven, Leuven, Belgium; Rheumatology Department, University Hospitals Leuven, Leuven, Belgium; Department of Development and Regeneration, KU Leuven, Leuven, Belgium

**Keywords:** interstitial lung disease, rheumatoid arthritis, epidemiology, prevalence

## Abstract

**Objectives:**

Interstitial lung disease (ILD) is an important comorbidity in RA. Its true prevalence remains unclear. We assessed reported prevalences across different diagnostic approaches.

**Methods:**

We conducted a systematic literature review and meta-analysis to assess RA-ILD prevalence. Embase, MEDLINE (PubMed), Cochrane Library and Web of Science were searched (from inception to 2022). Heterogeneity was assessed (*I*^2^). Subgroup differences were assessed (Q-test). Bias appraisal was performed via a funnel plot and Egger’s test. Individual studies were evaluated for the presence of bias using the AXIS score. Mixed effects meta-regression explored sample size, diagnostic modality, geography and AXIS score as moderators.

**Results:**

We included 74 studies and obtained an overall random effects pooled prevalence of 20.6% (95% CI 18.3, 23.2; *I*^2^ = 99.7%). The random effects pooled prevalence varied by diagnostic method: 31% (95% CI 25, 38; *I*^2^ = 97%) when using high-resolution CT, 21% (95% CI 14, 31; *I*^2^ = 95%) with pulmonary function tests, 13% (95% CI 5, 30; *I*^2^ = 99%) when combining multiple diagnostic tools, 12% (95% CI 7, 20; *I*^2^ = 96%) with chest X-ray and 5% (95% CI 4, 8; *I*^2^ = 100%) when based on International Classification of Diseases codes. Low-bias recent studies (AXIS >16; 2013–2022) yielded a pooled prevalence of 10.1% (95% CI 6.2, 16.3; *I*^2^ = 99.9%). Prevalences significantly differed between tools (Q = 73.16, df = 4, *P* < 0.0001). The meta-regression model (QM = 89.96, df = 11, *P* < 0.0001) had a moderate explanatory value (*R*^2^ = 47.82%). The asymmetric funnel plot (*t* = 2.59, df = 98, *P* = 0.011) pointed to significant bias (estimate of 4.86, SE = 1.88).

**Conclusion:**

Due to extreme heterogeneity and evidence of small-study effects, the overall pooled random effects prevalence (20%) should be interpreted cautiously and primarily reflects the wide variation in diagnostic approaches. Estimates derived from low-bias and multimodal diagnostic studies (≈10–13%) may provide a more clinically meaningful approximation, although they remain informed estimates rather than definitive prevalence rates.

Key messages

- The exact prevalence of interstitial lung disease (ILD) in RA remains uncertain due to extreme heterogeneity.
- The high residual heterogeneity after multivariate meta-regression underscores the need for standardized diagnostic criteria.
- Robust epidemiological research is needed to set up clinical practice and screening guidelines.

## Introduction

RA is a systemic autoimmune disease that typically presents with a symmetric polyarthritis. It is estimated that it affects ≈0.5–1% of the population, with varying geographical prevalence. Typically it presents in the fourth to sixth decade, with a female predominance. The diagnosis is made by an expert rheumatologist and is guided by the ACR/EULAR classification criteria [1]. Approximately 40% of patients with RA experience extra-articular disease manifestations [2]. One of the most frequently involved organs is the lung. In the lung, RA can cause pleuritis, vasculitis, pleural nodules, free-standing bronchiectasis and interstitial lung disease (ILD) [3].

ILD associated with RA (RA-ILD) is an extra-articular manifestation that has high morbidity and mortality. Typical symptoms consist of a dry cough and progressive dyspnoea upon exertion. Nevertheless, the presence of symptoms is not a sensitive diagnostic screening tool [4]. Pulmonary function tests typically show a restrictive pattern and reduced diffusion capacity. High-resolution CT (HRCT) scans most frequently show a usual interstitial pneumonia (UIP) pattern, but an organizing pneumonia (OP) or non-specific interstitial pneumonia pattern (NSIP) can also be present. The reported prevalence is currently unclear. Accurate prevalence estimates are critical for the development of efficient screening strategies and for understanding the disease burden.

The gold standard for the diagnosis of RA-ILD consists of a multidisciplinary discussion (MDD) encompassing the radiological, biochemical and clinical findings. A cornerstone for this MDD is the HRCT. The radiographic pattern will provide clues towards the underlying disease and allows for determination of the fibrotic extent. It also determines therapeutic choices. Furthermore, pulmonary function tests (PFTs) play an important role in the diagnosis and disease extent stratification [5]. Previously, thoracic X-ray was used in the diagnosis of ILDs. As it lacks sensitivity, the use of X-rays is no longer recommended in the diagnosis of ILDs [6]. However, some epidemiological studies have used chest X-rays in the past, as they are easily available and associated with lower radiation than HRCT. These studies are to be cautiously interpreted, as they might be reporting lower prevalences compared with the gold standard.

As the prevalence of RA-ILD is highly debated, this systematic review and meta-analysis aims to quantify the prevalence across different diagnostic methods and assess the impact of diagnostic variability.

## Methods

### Search strategy and data source

The aim of this systematic review was to assess the prevalence of ILD in patients with RA and to assess whether this prevalence differs when using different diagnostic approaches. The research question was formulated using a PICO (population, intervention, comparator, outcome) framework, with patients with RA as the population, diagnostic approaches for the detection of ILD as the index tests, comparisons between different diagnostic modalities and the primary outcome being the prevalence of ILD.

A comprehensive search strategy was developed using Medical Subject Heading (MeSH), synonyms and free text with various spellings. The search strategy was discussed with a medical research librarian (Learning Center Désiré Collen, KU Leuven, Leuven). Embase, MEDLINE (PubMed), Cochrane Library and Web of Science were searched from inception to 17 November 2022. All terms were adapted to the database used. The detailed search strategy can be found in Supplementary Files S1–2. Duplicates were removed using Endnote [7]. Articles were removed using the following sequence: 1. Web of Science (first removed), 2. Cochrane, 3. PubMed, 4. Embase. Ethical approval was not needed as this study only used previously published data. The review protocol was not registered in PROSPERO.

### Study selection

**Figure 1:**
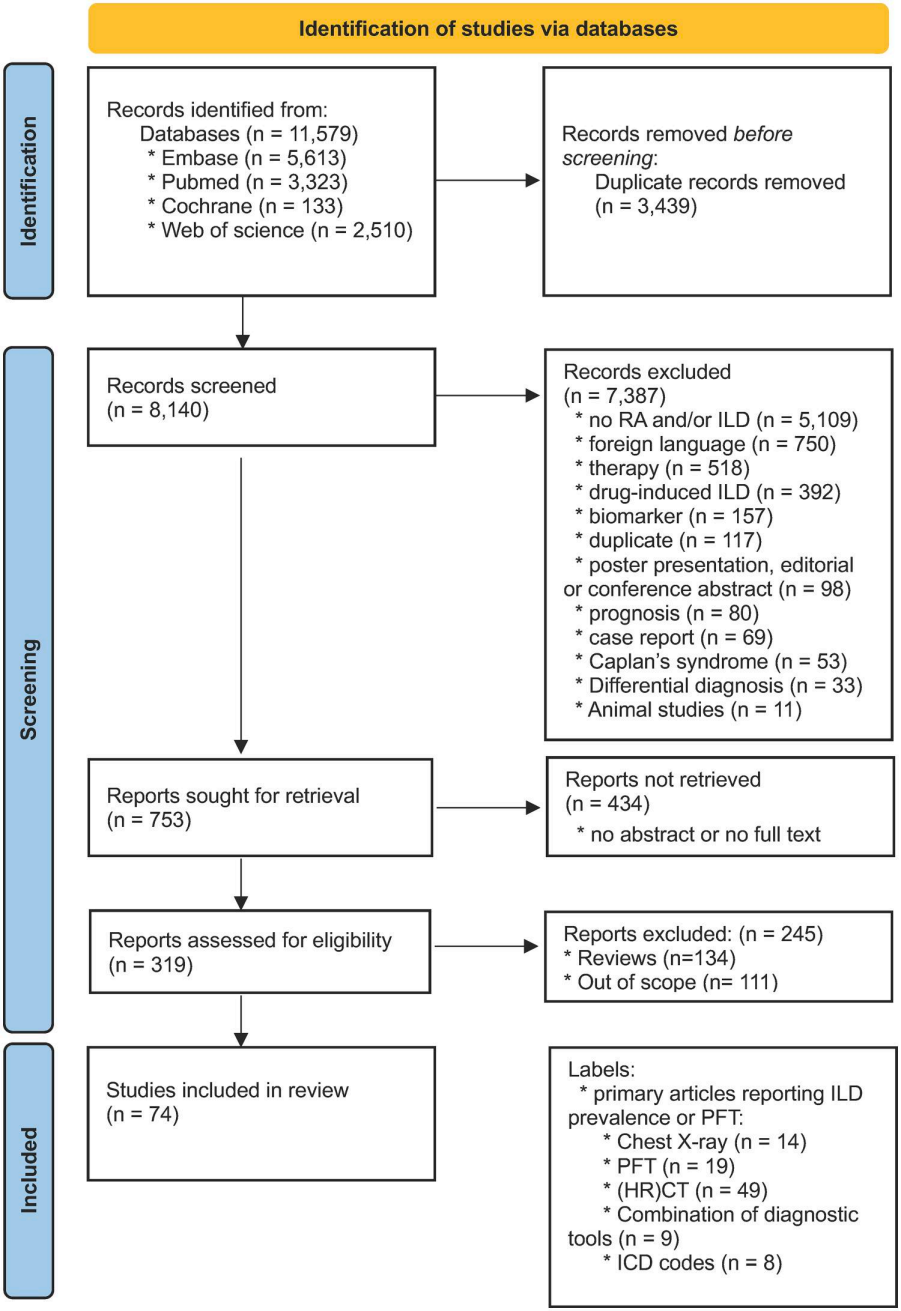
PRISMA flow diagram of the screening process. Included articles could receive multiple labels if different tools were separately used as screening tools

After study collection and deduplication, all studies were independently screened based on abstract by T.P. and M.V. using Rayyan (https://www.rayyan.ai/) [8]. We included original studies reporting the prevalence of ILD in adult RA patients using at least one diagnostic modality. Articles were excluded based on the following criteria: content being beyond the scope for this review, non-English literature, case reports or case series, remaining duplicates, editorials, conference abstracts and no abstract or full-text availability. The content of the included studies was assessed based on our predefined PICO question. The selection process is summarized in a Preferred Reporting Items for Systematic reviews and Meta-Analyses (PRISMA) [9] flow chart (Fig. 1).

### Data extraction

We extracted data from all primary studies that reported the prevalence of RA-ILD. The primary data extracted from the articles was the reported prevalence per diagnostic tool. As a primary outcome we report the pooled prevalence for RA-ILD for all included studies and all diagnostic tools included. As a secondary outcome we report the prevalence for RA-ILD per diagnostic tool. For all studies we also collected the author, publication year, total number of participants, number of patients with ILD, diagnostic tool used, whether the sample population was preselected, average RA disease duration, percentage of smokers, criteria used for the diagnosis of RA and criteria used for the diagnosis of ILD.

### Quality assessment

All studies were assessed for bias using the AXIS tool [10]. It was originally designed for cross-sectional studies, but it can also be used to assess longitudinal studies with prevalence as an outcome. The AXIS score is a critical appraisal tool in which a score from 0 to 20 is administered to every article. We stratified the articles into three groups: low bias (score 16–20), medium bias (12–15) and high bias (0–11). Furthermore, every article was assessed for the presence of possible patient selection bias.

### Data synthesis

#### Pooled prevalence and meta-analysis

We performed a meta-analysis of all the reported prevalences. However, in several primary studies, multiple prevalence estimates were derived from the same cohort using different diagnostic modalities [e.g. HRCT, PFT, chest X-ray or International Classification of Diseases (ICD)-based definitions]. Because these modality-specific estimates are not statistically independent, a conventional random effects model may underestimate uncertainty due to within-study clustering.

To account for this dependency structure, we performed a multilevel random effects meta-analysis using a hierarchical model in which diagnostic modality was nested within the study. This approach allows for the estimation of both between-study variability and additional within-study variability attributable to differences in diagnostic modality. Multilevel models were fitted using restricted maximum likelihood estimation of between-study variance (*τ*^2^).

In addition, as a sensitivity analysis, we restricted the dataset to a single prevalence estimate per cohort, prioritizing HRCT-based estimates when available, given their higher diagnostic sensitivity. Results from these alternative modelling approaches were compared to assess the robustness of the pooled prevalence estimates.

We also performed a random effects meta-analysis stratified per diagnostic tool: X-ray, HRCT, PFT, a combination of tools and ICD codes. A Q-test for subgroup differences was performed to evaluate whether pooled prevalences significantly differed across diagnostic tool categories. Forest plots were made per diagnostic tool. Additionally, we performed a meta-analysis excluding all high- and moderate-bias studies and excluding all studies published prior to 2012 to assess the pooled estimates in more recent, higher-quality studies.

All conventional random effects meta-analyses and meta-regression models were conducted on logit-transformed prevalence estimates, with between-study variance (*τ*^2^) estimated using the DerSimonian–Laird method.

#### Assessment of heterogeneity

We assessed overall heterogeneity and heterogeneity per diagnostic tool using *I*^2^ estimates. *I*^2^ is expressed as a percentage and represents the proportion of total variability that is due to heterogeneity. An *I*^2^ >75% was high. An *I*^2^ of 25–50% was consistent with a moderate degree of heterogeneity and an *I*^2^ <25% would be in line with a low degree of heterogeneity.

To assess the presence of small-study effects and publication bias, we constructed funnel plots of the logit-transformed prevalence estimates against their standard errors and conducted Egger’s regression tests for funnel plot asymmetry [11].An α < 0.05 was considered indicative of asymmetry.

To explore potential sources of heterogeneity, we conducted univariate and mixed effects multivariate meta-regression using the DerSimonian–Laird estimator for *τ*^2^ [12]. *τ*^2^ is the estimated variance of the true effect size across studies. Moderators included total sample size, year of publication, the used diagnostic tool, geographical differences per continent and the AXIS score. Geographical differences were included, as they capture genetic differences and many potential environmental and socio-economic differences. The outcome variable for all the models were logit-transformed prevalences.

All statistical analyses were performed in R version 4.5.0 )R Foundation for Statistical Computing, Vienna, Austria) using the meta [13] and metafor [14] packages.

## Results

### Study selection and quality assessment

We identified a total of 11 579 articles, of which 5613 were found via Embase, 3323 via PubMed, 133 via Cochrane and 2510 via Web of Science. We removed 3439 duplicates after deduplication. A total of 8140 articles were screened based on title and abstract. Based on the aforementioned selection criteria, we identified a total of 314 relevant abstracts. After further full-text assessment we identified 74 relevant articles. Of these, 17 articles were scored as high bias, 34 articles were scored as moderate bias and 23 articles were scored as low bias. The screening process is shown as a PRISMA flow chart (Fig. 1). A total of 14 studies contained prevalences based on the use of chest X-ray, 19 articles contained prevalences based on PFTs, 49 used HRCT, 9 opted for a combination of diagnostic tools and 9 included prevalences based on ICD codes. One study used a prospective set-up, while all other studies were either retrospective or cross-sectional.

### Study characteristics

Pooling all reported prevalences and using a random effects meta-analysis to account for the extreme heterogeneity between studies, we obtained a prevalence of 20.6% (95% CI 18.3, 23.2; *I*^2^ = 99.7%). A sensitivity analysis restricting each cohort to a single prevalence estimate (prioritizing HRCT-based estimates when available) yielded a pooled prevalence of 21.7% (95% CI 16.7, 27.6; I^2^ = 99.8%).

Furthermore, a multilevel random effects model accounting for clustering of diagnostic modalities within studies produced a pooled prevalence of 20.7% (95% CI 16.4, 25.8). The similarity of estimates across modelling approaches indicates that within-study dependence did not materially influence the overall prevalence estimate.

**Figure 2:**
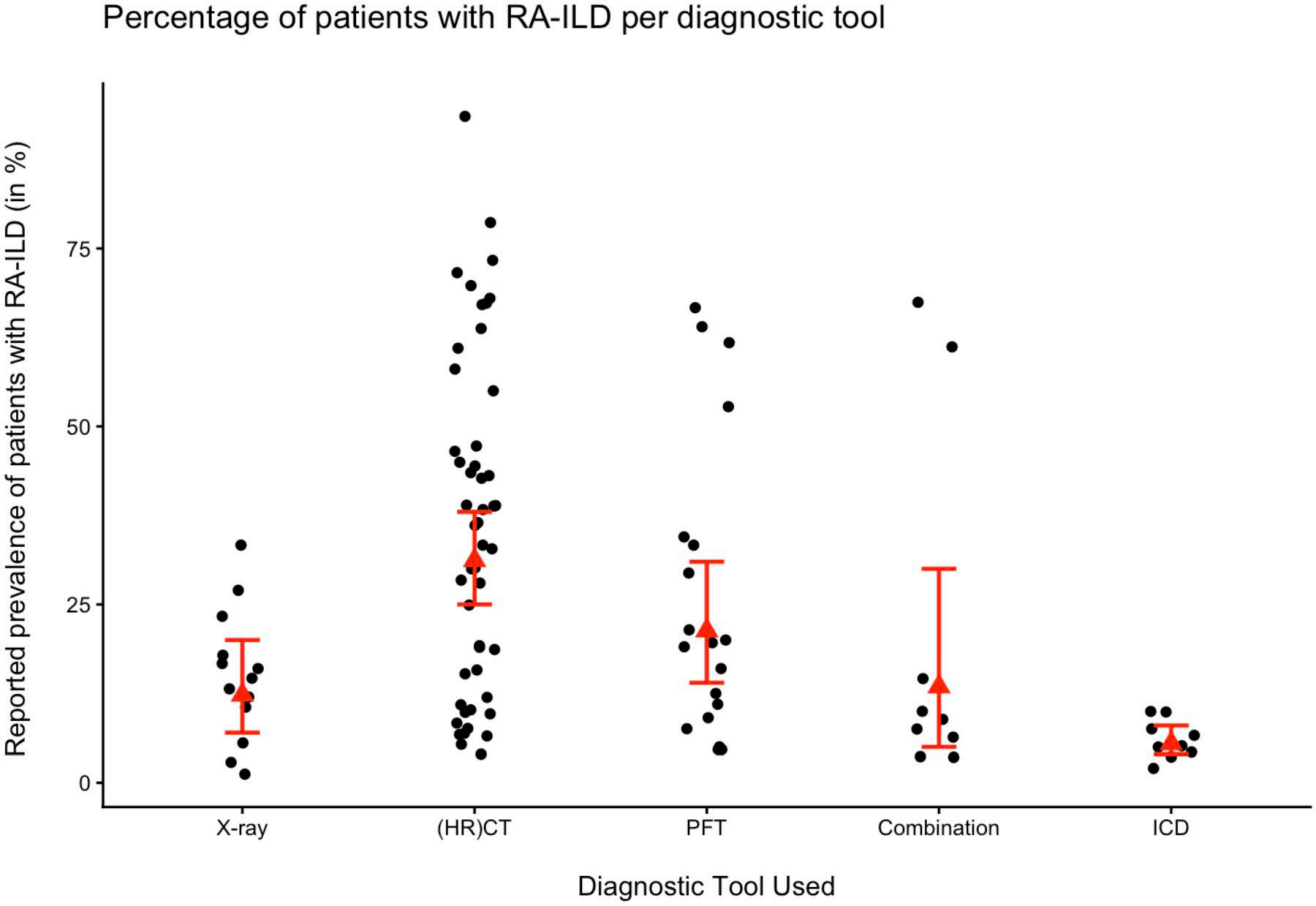
The reported prevalence of every included study (black dots) per diagnostic tool. Per diagnostic tool, the random effect pooled prevalences are presented as a red triangle, with the red line signifying the 95% CI

The meta-analysis included a total of 98 979 events for a total of 1 487 575 observations. Fig. 2 shows an overview of all reported prevalences and all random effects pooled prevalences per diagnostic tool. Pooled prevalences significantly varied based on the diagnostic tool used (Q = 73.16, df = 4, *P* < 0.0001).

### Results per diagnostic tool

#### X-ray

**Figure 3:**
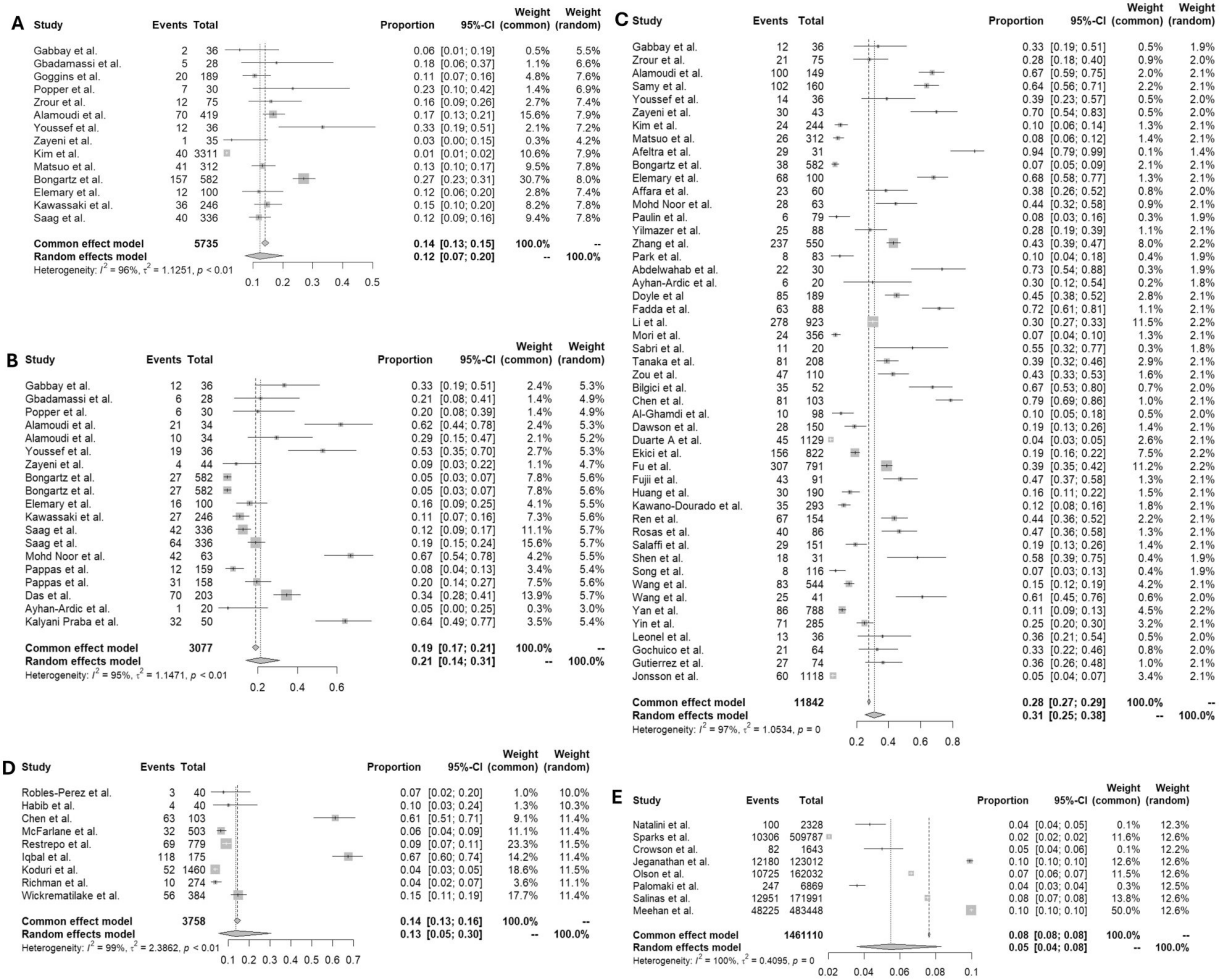
(A) Forest plot illustrating the prevalence of ILD in patients with RA when using X-ray as a diagnostic tool. **(B)** Forest plot illustrating the prevalence of ILD in patients with RA when using PFTs as a diagnostic tool. **(C)** Forest plot illustrating the prevalence of ILD in patients with RA when using HRCT as a diagnostic tool. **(D)** Forest plot illustrating the prevalence of ILD in patients with RA when using a combination of diagnostic tools. **(E)** Forest plot illustrating the prevalence of ILD in patients with RA when using ICD codes

Fourteen articles that used X-ray as a diagnostic tool to detect ILD were included in this analysis. The random effects model showed a pooled prevalence of ILD of 12% (95% CI 7, 20). Fig. 3A shows the forest plot for all included studies. The meta-analysis showed high heterogeneity (*I*^2^ = 96%). The different studies included different definitions and classification criteria for RA. Additionally, terminology used to describe radiographic abnormalities differed. Some studies referred specifically to ‘interstitial lung disease’, while others used broader terms such as ‘interstitial abnormalities’, often without clear definitions. Table 1A shows the overview of the included studies, RA classification criteria used, terminology used to describe X-ray abnormalities, publication year, prevalence of ILD, number of patients screened, percentage of female patients, median RA duration and qualitative bias assessment.

**Table 1:** Study characteristics per diagnostic tool.

Study characteristics for studies using X-ray to detect ILD											
Author	Publication year	Country	Definition of RA	Definition of ILD	Prevalence of ILD, %	Patients screened, *n*	Female, %	RA duration, median, years	Former or current smoker, %	AXIS score	Sample preselection
Gabbay *et al.* [25]	1997	Australia	1987 ACR	ILD	5.55	36	69.44	1.1	55.56	16	No
Gbadamassi *et al.* [26]	2020	Togo	2010 ACR/EULAR	ILD	17.86	28	85.71	4.1	NA	13	No
Goggins *et al.* [27]	2019	Ireland	2010 ACR/EULAR	ILD	10.58	189	72.22	16	55.05	14	Yes, not all patients received X-ray
Popper *et al.* [28]	1972	USA	1958 ARA	ILD	23.33	30	66.67	NA	46.67	8	No
Zrour *et al.* [29]	2005	Tunisia	1987 ACR	ILD	16	75	84.00	8	14.67	12	No
Alamoudi *et al.* [30]	2017	Saudi Arabia	ICD and 2010 ACR/EULAR	Abnormalities	16.70	419	86.87	3.5	15.95	7	Yes, unclear whether all patients received all exams
Youssef *et al.* [31]	2012	Egypt	1987 ACR	Abnormalities	33.33	36	94.44	8	0.00	10	Yes, unclear study design
Zayeni *et al.* [32]	2016	Iran	2010 ACR/EULAR	Abnormalities	2.85	35	79.55	NA	0.00	8	Yes, only non-smokers and asymptomatic patients
Kim *et al.* [33]	2017	Republic of Korea	1987 ACR	ILD	1.21	3311	84.25	14.9	16.65	15	Yes, only on clinical indication
Matsuo *et al.* [34]	2019	Japan	NA	ILD	13.14	312	86.86	14.9	30.45	16	No
Bongartz *et al.* [35]	2010	USA	1987 ACR	Abnormalities	23.98	582	73.37	16.4	52.92	17	Yes, not all patients received X-ray
Elemary *et al.* [36]	2021	Egypt	2010 ACR/EULAR	Abnormalities	12	100	77.00	14.9	65.00	10	No
Kawassaki *et al.* [37]	2015	Brazil	1987 ACR	Abnormalities	14.63	246	84.96	16	51.22	14	No
Saag *et al.* [38]	1996	USA	1987 ACR	Abnormalities	11.90	336	70.24	13.9	NA	16	No

Study characteristics for studies using PFTs to detect ILD												
Author	Publication year	Country	Definition of RA	PFT definition used	PFT cut-off used	Prevalence of ILD	Patients screened, *n*	Female, %	RA duration, median, cyears	Former or current smokers, %	AXIS score	Sample preselection
Gabbay *et al.* [25]	1997	Australia	1987 ACR	Decreased DLCO	DLCO <75%	33.33	36	69.44	1.10	55.56	16	No
Gbadamassi *et al.* [26]	2020	Togo	2010 ACR/EULAR	Restrictive lung pattern	Not defined	21.43	28	85.71	4.10	NA	13	No
Popper *et al.* [28]	1972	USA	1958 ARA	Decreased DLCO	DLCO <80%	20.00	30	NA	NA	46.67	8	No
Alamoudi *et al.* [30]	2017	Saudi Arabia	ICD and 2010 ACR/EULAR	Restrictive lung pattern	Not defined	61.76	34	86.87	3.50	15.95	7	Yes, unclear whether all participants received all tests
Alamoudi *et al.* [30]	2017	Saudi Arabia	ICD and 2010 ACR/EULAR	Decreased DLCO	Not defined	29	34	86.87	3.50	15.95	7	Yes, unclear whether all participants received all tests
Youssef *et al.* [31]	2012	Egypt	1987 ACR	Restrictive lung pattern	FVC %pred <80%	52.78	36	94.44	8.00	0.00	10	No
Zayeni et al. [32]	2016	Iran	2010 ACR/EULAR	Restrictive lung pattern	Not defined	9.09	44	79.55	NA	0.00	8	Yes, only asymptomatic non-smokers included
Bongartz *et al.* [35]	2010	USA	1987 ACR	Restrictive lung pattern	TLC %pred <80%	4.64	582	73.37	16.40	52.92	17	No
Bongartz *et al.* [35]	2010	USA	1987 ACR	Decreased TLC	TLC %pred <80%	4.64	582	73.37	16.40	52.92	17	No
Elemary *et al.* [36]	2021	Egypt	2010 ACR	Restrictive lung pattern	Not defined	16.00	100	77.00	14.90	65.00	10	No
Kawassaki *et al.* [37]	2016	Brazil	1987 ACR	Restrictive lung pattern	FEV1:FVC ratio ≥LLN+FVC <LLN and no post-bronchodilator normalization of FVC	10.98	246	84.96	16	51.22	14	No
Saag *et al.* [38]	1996	USA	1987 ACR	Decreased FVC	FVC %pred < 80%	13	336	70.24	13.90	NA	16	No
Saag *et al.* [38]	1996	USA	1987 ACR	Decreased DLCO	DLCO <80%	19.05	336	70.24	13.90	NA	16	No
Mohd Noor *et al.* [39]	2009	Malaysia	1987 ACR	Restrictive lung pattern	TLC %pred <95%, FVC %pred <95%, FEV1 %pred <95% and FEV1:FVC ratio >75%	66.67%	63	88.89	14.00	0.0634921	15	No
Pappas *et al.* [40]	2010	USA	1987 ACR	Restrictive lung pattern	FVC %pred <80% with no signs of obstruction	8	159	61.01	10.70	57.86	14	No
Pappas *et al.* [40]	2010	USA	1987 ACR	Decreased DLCO	DLCO %pred <80%, with no signs of obstruction	19.62	158	61.01	10.70	57.86	14	No
Das *et al.* [41]	2020	India	1987 ACR	Restrictive lung pattern	FVC <LLN, FEC1:FVC ratio ≥LLN	34.48	203	87.19	7.10	0	13	No
Ayhan-Ardic *et al.* [42]	2006	Turkey	1987 ACR	Restrictive lung pattern	Decreased FVC and FEV1, normal FEV1:FVC ratio	5	20	75.00	11.10	NA	15	No
Kalyani Praba *et al.* [43]	2017	India	2010 ACR	Restrictive lung pattern	FEV1:FVC ratio >70% and reduced VC and TLC	64.00	50	85.00	NA	0.00	5	No

Study characteristics for studies using HRCT to detect ILD											
Author	Publication year	Country	Definition of RA	Definition of HRCT findings	Prevalence of ILD, %	Patients screened, *n*	Female, %	RA duration, median, years	Former or current smokers, %	AXIS score	Sample preselection
Gabbay *et al.* [25]	1997	Australia	1987 ACR	ILD	33.33	36	69.44	1.10	44.44	16	No
Zrour *et al.* [29]	2005	Tunisia	1987 ACR	ILD	28.00	75	84.00	8.00	85.33	12	No
Alamoudi *et al.* [30]	2017	Saudi Arabia	ICD and 2010 ACR/EULAR	CT abnormalities	67.11	149	86.87	3.50	84.05	7	No
Samy *et al.* [44]	2021	Egypt	2010 ACR/EULAR	CT abnormalities	63.75	160	85.00	4.98	85.00	8	No
Youssef *et al.* [31]	2012	Egypt	1987 ACR	ILD	39	36	94.44	8.00	100.00	10	No
Zayeni *et al.* [32]	2016	Iran	2010 ACR/EULAR	CT abnormalities	69.77	43	79.55	NA	100.00	8	Yes, limited to asymptomatic former smokers
Kim *et al.* [33]	2017	Republic of Korea	1987 ACR	ILD	9.84	244	84.25	NA	83.35	15	No
Matsuo *et al.* [34]	2020	Japan	NA	ILD	8.33	312	86.86	14.90	69.55	16	No
Afeltra *et al.* [45]	2006	Italy	1987 ARA	CT abnormalities	93.55	31	90.32	NA	NA	9	Yes, exams were performed on a clinical indication
Bongartz *et al.* [35]	2010	USA	1987 ACR	CT abnormalities	6.53	582	73.37	16.40	47.08	17	No
Elemary *et al.* [36]	2021	Egypt	2010 ACR	CT abnormalities	68.00	100	77.00	14.90	35.00	10	No
Affara *et al.* [46]	2016	Egypt	2010 ACR	ILD	38	60	71.67	NA	71.67	11	Yes, only patients treated with MTX and NSAIDs were included, no patients using bDMARDs
Mohd Noor *et al.* [39]	2009	Malaysia	1987 ACR	ILD	44.44	63	88.89	14.00	93.65	15	No
Paulin *et al.* [47]	2021	Argentina	2010 ACR/EULAR	HRCT abnormalities	7.59	79	83.13	0.25	49.39	13	Yes, early RA
Yilmazer *et al.* [48]	2016	Turkey	1987 ACR	HRCT abnormalities defined as ground glass, pleural irregularities, honeycombing, septam/subpleural lines, nodules, bronchiectasis	28	88	70.77	9.32	66.92	12	Yes, exams were performed on a clinical indication
Zhang *et al.* [49]	2017	China	1987 ARA or 2010 ACR/EULAR	ILD	43.09	550	70.00	8.00	NA	12	No
Park *et al.* [50]	2016	Republic of Korea	2010 RA classification criteria	HRCT abnormalities	9.64	83	NA	NA	NA	15	No
Abdelwahab *et al.* [51]	2022	Egypt	2010 ACR, EULAR	ILD	73	30	86.67	0.5833333	33.33	12	Yes, early RA cohort
Ayhan-Ardic *et al.* [42]	2006	Turkey	1987 ARA	ILD	30.00	20	75.00	11.10	NA	15	No
Doyle et al. [52]	2015	USA	ACR criteria at time of inclusion in cohorts	ILD	44.97	189	NA	NA	NA	18	Yes, exams were performed on a clinical indication
Fadda *et al.* [53]	2018	Egypt	2010 ACR/EULAR	ILD	72	88	85.23	10.20	98.86	10	Yes, exams were performed on a clinical indication
Li *et al.* [54]	2020	China	1987 ACR or 2010 ACR/EULAR	ILD	30.12	923	72.59	6.00	74.00	16	Yes, exams were performed on a clinical indication
Mori *et al.* [55]	2012	Japan	1987 ACR	ILD	6.74	356	74.85	NA	77.61	17	Yes, exams were performed on a clinical indication
Sabri *et al.* [56]	2016	Egypt	NA	ILD	55.00	20	70.00	NA	NA	2	Yes, only symptomatic patients
Tanaka *et al.* [57]	2022	Japan	1987 ACR or 2010 ACR/EULAR	ILD	38.94	208	69.23	7.94	NA	16	Yes, only patients using TNF blockers
Zou *et al.* [58]	2012	China	1987 ACR	ILD	42.73	110	71.82	8.25	82.73	11	No
Bilgici *et al.* [59]	2005	Turkey	1987 ARA	CT abnormalities defined as grade 1: ground-glass opacities; grade 2: reticular pattern; and grade 3: reticulonodular (fibrotic) lesion pattern	67.31	52	84.62	8.37	25.00	16	No
Chen *et al.* [60]	2013	China	1987 ACR	CT abnormalities defined as the presence/absence of ground-glass opacification, septal thickening, reticulation, traction bronchiectasis, and/or honeycombing	79	103	73.79	4.30	NA	13	No
Al-Ghamdi *et al.* [61]	2009	Saudi Arabia	1987 ACR	ILD	10.20	98	75.51	NA	96.97	5	Yes, unclear whether everyone received all exams
Dawson *et al.* [62]	2001	UK	1987 ACR	ILD	18.67	150	66.67	12.70	0.3	17	No
Duarte *et al.* [63]	2019	UK	NA	ILD	4	1129	NA	NA	NA	16	Yes, exams were performed on a clinical indication
Ekici *et al.* [64]	2021	Turkey	2010 ACR/EULAR	ILD	18.98	822	NA	NA	NA	17	Yes, exams were performed on a clinical indication
Fu *et al.* [65]	2019	China	1987 ACR or 2010 ACR/EULAR	ILD	38.81	791	NA	NA	NA	13	No
Fujii *et al.* [66]	1993	Japan	ACR, year not specified	ILD	47	91	NA	NA	NA	11	No
Huang *et al.* [67]	2020	USA	1987 ACR or 2010 ACR/EULAR	ILD	15.79	190	80.00	21.20	40.00	14	Yes, exams were performed on a clinical indication
Kawano-Dourado *et al.* [68]	2020	Brazil	2010 ACR/EULAR	ILD	11.95	293	82.46	NA	66.79	19	Yes, exams were performed on a clinical indication
Ren *et al.* [69]	2022	China	2010 ACR/EULAR	ILD	44	154	0.00	2.00	23.38	15	Yes, preselected to males and smokers
Rosas *et al.* [70]	2011	USA	1987 ACR	ILD	46.51	86	76.74	NA	53.49	14	No
Salaffi *et al.* [71]	2019	Italy	2010 ACR	ILD	19.21	151	70.20	7.50	76.16	11	Yes, exams were performed on a clinical indication
Shen *et al.* [72]	2011	China	1987 ACR	ILD	58.06	31	83.87	NA	NA	12	No
Song *et al.* [73]	2016	South Korea	1987 ACR	ILD	6.90	116	NA	NA	NA	15	No
Wang *et al.* [74]	2015	China	2006 ACR	ILD	15.26	544	78.49	NA	NA	13	No
Wang *et al.* [75]	2015	China	1987 ACR	ILD	60.98	41	51.22	NA	NA	14	No
Yan *et al.* [76]	2019	China	1987 ACR	ILD	11	788	77.03	4.00	NA	14	Yes, exams were performed on a clinical indication
Yin *et al.* [77]	2014	China	1987 ACR	ILD	24.91	285	74.04	5.00	79.30	14	Yes, exams were performed on a clinical indication
Leonel *et al.* [78]	2012	Mexico	1987 ACR	CT abnormalities	36.11	36	100.00	NA	NA	11	Yes, only female RA patients
Gochuico *et al.* [79]	2008	USA	1987 ACR	ILD	32.81	64	83.72	11.30	46.51	13	Yes, cohort was pooled with patients who were known with RA-ILD
Gutierrez *et al.* [80]	2022	Mexico	2010 ACR/EULAR	ILD, based on Warrick score	36.49	74	86.49	8.03	NA	17	No
Jönsson *et al.* [81]	2021	Sweden	1987 ACR	ILD	5.37	1118	67.71	NA	40.43	19	Yes, HRCT was not performed if an X-ray was diagnostic and it was an early RA cohort

Study characteristics for studies using a multimodal detection approach to detect ILD											
Author	Publication year	Country	Definition of RA	Detection method	Prevalence of ILD, %	Patients screened, *n*	Female, %	RA duration, median, years	Former or current smokers, %	AXIS score	Sample preselection
Robles-Pérez *et al.* [82]	2020	Spain	1987 ACR	X-ray + PFT + HRCT	7.50	40	NA	NA	59.38	15	No
Habib *et al.* [83]	2011	Egypt	1987 ACR	PFT + HRCT	10.00	40	70.00	18	12.5	15	No
Chen *et al.* [60]	2013	China	1987 ACR	PFT + HRCT	61.17	103	73.79	4.3	NA	13	No
McFarlane *et al.* [84]	2019	USA	2010 ACR	PFT + HRCT	6.36	503	87.87	NA	29.42	13	No
Restrepo *et al.* [85]	2015	USA	1987 ARA	Medical chart review (chest X-ray, PFT, CT, biopsy)	9	779	74.53	NA	56.49	16	Yes, exams were performed on a clinical indication
Iqbal *et al.* [86]	2022	Pakistan	NA	X-ray, PFT, CT, biopsy	67.43	175	75.43	6.2^a^	NA	15	No
Koduri *et al.* [87]	2010	UK	ACR, year not specified	Chest X-ray, PFT, HRCT, autopsy	3.56	1460	66.37	NA	40.90	17	No
Richman *et al.* [88]	2013	USA	1987 ACR	Medical chart review (HRCT, PFT (DLCO), CT, dyspnoea or crackles)	3.65	274	86.50	6.4	29.93	13	No
Wickrematilake *et al.* [89]	2021	Sri Lanka	1987 ARA or 2010 ACR/EULAR	Chest X-ray, clinical characteristics, HRCT	14.58	384	NA	NA	NA	7	Yes, exams were performed on a clinical indication

Study characteristics for studies using ICD codes to estimate the prevalence of ILD											
Author	Publication year	Country	Definition of RA	ICD codes used	Prevalence of ILD, %	Patients screened, *n*	Female, %	RA duration, median	Former or current smokers, %	AXIS score	Sample preselection
England *et al.* [90]	2018	USA	1987 ACR	ICD-9: 515–517, 714.81; ICD-10: M05.1x, J84.1x, J84.2, J84.89, J84.9, J99	5.16	2053	9.74	NA	79.06	18	Yes, Veterans Affairs RA registry
Natalini *et al.* [91]	2021	USA	1987 ACR	ICD-9: 515–517, 714.81; ICD-10: M05.1x, J84.1x, J84.2, J84.89, J84.9, J99	4.30	2328	11.30	8.00	0.790 718	16	Yes, Veterans Affairs RA registry
Sparks *et al.* [92]	2021	USA	Two or more ICD-9 or ICD-10 codes for ILD	Two or more ICD-9 or ICD-10 codes for ILD	2.02	509 787	76.22	NA	16.70	16	Yes, Medicare-based registry
Crowson *et al.* [93]	2022	USA	1987 ACR	ICD-9 515, 516.3x, 516.8, 516.9, 714.81, 714.89; ICD-10: J84.1x, J84.2, J84.09, J84.9, M05.1x, M06.4	4.99	1643	71.58	6.50	NA	16	No
Jeganathan *et al.* [94]	2021	USA	ICD-10: M05.1–M06.9	ICD-10 codes M05.1/J99.0, J84.1 and J84.9	10	123 012	73.65	NA	NA	17	No
Olson *et al.* [95]	2011	USA	ICD-9: 714.0–714.9 (excluding codes 714.3 and 714.4); ICD-10: M05.0–M06.9	ICD-9: 714.8, 515 and 516.3; ICD-10: M05.1, J99.0 and J84.1	6.62	162 032	NA	NA	NA	16	No
Palomäki *et al.* [96]	2021	Finland	ICD-10: M05 or M06	ICD-10: J84 and M05.1/J99.0; ICD-9: 515 and 516; ICD-8: 484.99 or 517.01	3.60	6869	71.10	NA	NA	20	No
Salinas *et al.* [97]	2021	USA	ICD-10: M05 or M06	ICD-10: J84	7.53	171 991	NA	NA	NA	15	No
Meehan *et al.* [98]	2022	USA	ICD-9-CM: 714.0 or 714.2	List of ICD-9-CM and ICD-10-CM codes	9.98	483 448	NA	NA	NA	13	Yes, needed to be seen by pulmonology before receiving ICD code for ILD and Medicare-based registry

ARA: American Rheumatism Association; %pred: percentage of predicted; LLN: lower limit of normal.

a The average duration was reported instead of the median duration.

#### PFT

Nineteen articles used PFTs to determine the prevalence of RA-ILD. In addition to the different RA classification criteria, different physiological criteria were used to determine the presence of ILD. The studies also used different physiological definitions based on the following pulmonary function values: forced vital capacity (FVC), diffusion capacity of the lung for carbon dioxide (DLCO), forced expiratory volume in 1 s (FEV1), total lung capacity, FEV1:FVC (the ratio between FEV1 and FVC, expressed as a percentage), total lung capacity (TLC) and vital capacity (VC). Table 1B presents an overview of the included studies, including the RA classification criteria used, PFT definition, physiological cut-off used, publication year, prevalence of ILD, number of patients screened, percentage of female patients, median RA duration and qualitative bias assessment. Fig. 3B shows the forest plot for all included studies. The random effects model showed a prevalence of 21% (95% CI 14, 31). The meta-analysis showed very high heterogeneity (*I*^2^ = 95%).

#### HRCT scan

Forty-nine articles used HRCT scans to determine the prevalence of RA-ILD. Some papers labelled HRCT findings as the presence or absence of ILD, others described whether any HRCT abnormalities were present. Table 1C incorporates an overview of the included studies, including the RA classification criteria used, terminology to describe CT findings, publication year, prevalence of ILD, number of patients screened, percentage of female patients, median RA duration and qualitative bias assessment. The random effects pooled prevalence was 31% (95% CI 25, 38). The forest plot is shown in Fig. 3C. The heterogeneity of the meta-analysis was high (*I*^2^ = 97%).

#### Combination of multiple diagnostic tools

Nine studies employing a combination of diagnostic approaches—including chest X-ray, PFTs and HRCT—to detect ILD in patients with RA were included in this analysis. The pooled prevalence of ILD based on the random effects model was 13% (95% CI 5, 30), as illustrated in Fig. 3D. The included studies exhibited very high heterogeneity (*I*^2^ = 99%), likely reflecting variations in diagnostic criteria, imaging modalities and patient selection. While all studies used a combination of imaging and functional testing, the extent and rationale for testing differed, with some studies applying imaging selectively based on clinical indications. Definitions of RA varied, with most using the 1987 ACR criteria, although some adopted more recent or unspecified definitions. Sample characteristics also differed significantly, including RA duration and smoking status, both of which may influence ILD prevalence. Table 1D summarizes the characteristics of each study, including RA classification criteria, diagnostic modalities, ILD prevalence, patient demographics and risk of bias assessment.

#### ICD code

Nine studies used diagnostic coding (ICD-9 and/or ICD-10) to define both RA and ILD in administrative datasets. The pooled prevalence of ILD across these studies was 5% (95% CI 4, 8) based on a random effects model (Fig. 3E), with extreme heterogeneity (*I*^2^ = 100%). This likely reflects differences in coding practices, reimbursement practices, case definitions, population characteristics and sample selection criteria. Most studies were conducted in the USA, with one from Finland. RA was variably defined using ACR classification criteria or via diagnostic coding alone, while ILD case ascertainment generally relied on a broad range of pulmonary ICD codes. Several studies used large national or insurance-based registries. Reporting of demographic and clinical characteristics was inconsistent; female representation varied widely and information on RA duration and smoking history was frequently missing. Table 1E summarizes the characteristics and methodological quality (based on AXIS scores) of the included studies.

### Low-bias analysis, 2013–2022

Eighteen articles were found when including all articles that were published between 1 January 2013 and 31 December 2022 and had an AXIS score ≥16. One study used a combination of methods, 1 used X-ray, 10 used HRCT and 6 used ICD codes. When performing the meta-analysis combining all low-bias studies, we obtained a pooled prevalence of 10.1% (95% CI 6.2, 16.3) using a random effects model. The heterogeneity of this model remained very high (*I*^2^ = 99.9%). The forest plot of this analysis can be found in Supplementary File 2.

### Studies using multiple diagnostic tools

Thirteen studies reported prevalence using more than one diagnostic modality within the same population. Across the 12 cohorts that reported both HRCT- and X-ray-based prevalences, HRCT yielded significantly higher prevalence estimates than X-ray (paired Wilcoxon signed-rank test: *V* = 21, one-sided *P* = 0.016). The median within-cohort increase in prevalence with HRCT compared with X-ray was +39.1 percentage points, with differences ranging from +5.6 to +66.9 percentage points.

### Assessment of heterogeneity

**Figure 4:**
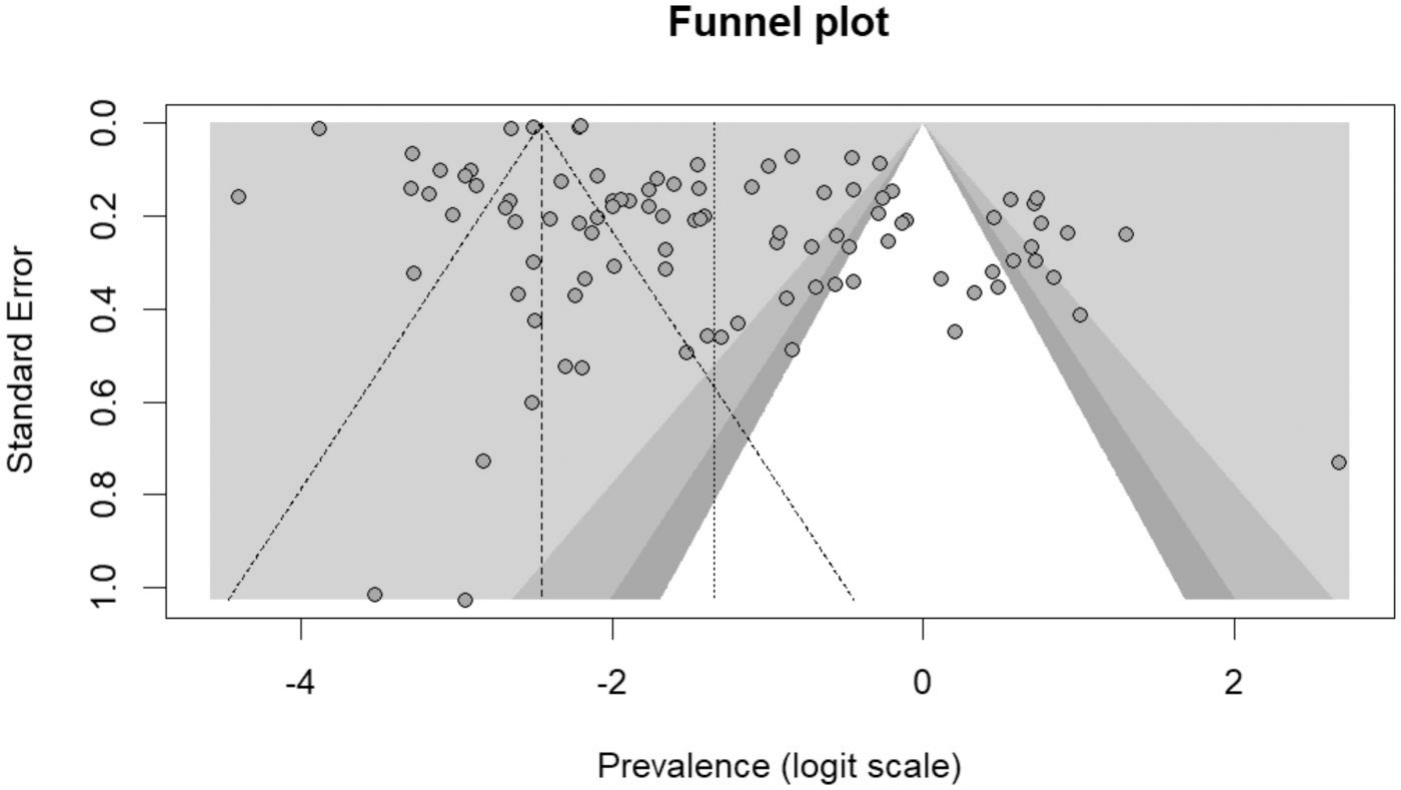
Funnel plot assessing potential publication bias in the included studies. A funnel plot is a scatter plot where study effect size is plotted on the horizontal axis and the standard error is plotted on the vertical axis. A symmetrical distribution of points suggests a low risk of bias, while asymmetry may indicate potential publication bias or small-study effects

We observed very high *I*^2^ values, and therefore very high measures of heterogeneity, for all performed meta-analyses. Visual inspection of the funnel plot revealed marked asymmetry (Fig. 4), suggesting possible small-study effects. Egger’s regression test confirmed significant asymmetry (*t* = 2.59, df = 98, *P* = 0.011), with a bias estimate of 4.86 (s.e. = 1.88).

In univariate meta-regression, study sample size (*P* = 0.0002), geographic region (*P* < 0.0001), diagnostic modality (*P* = 0.0011) and AXIS score (<0.0001) were significantly associated with RA-ILD prevalence, while year of publication was not (*P* = 0.17). A mixed effects meta-regression model including sample size, geographic variation, diagnostic modality and AXIS score as covariates was statistically significant (QM = 89.96, df = 11, *P* < 0.0001). Eight studies were omitted for model fitting due to missing data. The model explained 47.82% (*R*^2^) of the between-study heterogeneity. Substantial residual heterogeneity remained after meta-regression (*τ*^2^ = 0.69, *I*^2^ = 94.98%). In the multivariable model, study sample size (*P* < 0.0001) and AXIS score (*P* = 0.037) remained independently associated with RA-ILD prevalence, while geographic differences were not. Larger sample sizes and higher bias, expressed by a lower AXIS score, were associated with lower prevalence. Compared with studies using HRCT, those using chest X-ray (β = −0.88, *P* = 0.002), PFTs (β = −0.56, *P* = 0.028) or diagnostic tool combinations (β = −0.68, *P* = 0.039) reported significantly lower prevalence. Studies using ICD codes did not independently differ significantly from those using HRCT when using this multivariate model.

## Discussion

A significant difference in prevalence was observed across diagnostic modalities (*P* < 0.001). The random effects pooled prevalence of ILD was highest for HRCT [31% (95% CI 25, 38)] and lowest when using ICD codes [5% (95% CI 4, 8)] when based on ICD codes. In the overall multilevel random effects model accounting for clustering of diagnostic modalities within studies, the pooled prevalence was 20.7% (95% CI 16.4, 25.8). As anticipated, heterogeneity was exceptionally high in both the primary and sensitivity analyses (*I*^2^ > 99%), with similarly high heterogeneity within all diagnostic subgroups (*I*^2^ = 95–100%). Funnel plot asymmetry (*P* = 0.011) suggested small-study effects, indicating that the overall pooled estimate may be inflated. In addition, several studies demonstrated potential selection bias, such as inclusion of patients undergoing CT imaging based on clinical suspicion rather than systematic screening. These factors necessitate caution in interpreting the overall pooled prevalence.

Estimates derived from low-bias and multimodal diagnostic studies (≈10–13%) likely provide a more clinically applicable approximation of RA-ILD prevalence than the overall pooled estimate. However, even these estimates should be interpreted cautiously, as substantial residual heterogeneity, potential small-study effects and selection bias may still contribute to overestimation. Accordingly, these figures are best viewed as informed approximations rather than definitive prevalence rates for guideline development or clinical trial design.

Importantly, diagnostic modalities should not be interpreted as equivalent measurement constructs. HRCT is more sensitive for early and subclinical ILD, whereas chest X-ray may substantially underestimate disease, which is also highlighted by the studies that assessed multiple diagnostic tools in the same cohort. PFT- and ICD-based definitions capture different constructs, namely functional impairment and administrative coding, rather than radiologically confirmed ILD. Diagnostic modality was therefore included as a subgroup variable to explore heterogeneity rather than to imply equivalence. Formal adjustment for diagnostic sensitivity was not feasible because sensitivity and specificity data were inconsistently reported across studies.

### Meta-regression analysis

Using a univariate meta-regression, publication year was not a significant moderator (*P* = 0.17), suggesting that the observed heterogeneity is unrelated to temporal trends. Multivariate meta-regression showed that lower prevalence was observed in studies with a higher AXIS score and in studies with larger sample sizes (*P* < 0.0001, β = −0.0011). Studies using ICD coding tended to have large sample sizes, suggesting that the initially observed lower prevalence in this subgroup may be confounded by study size rather than reflecting an independent effect of the diagnostic method. Nevertheless, as ICD coding is often linked to medication prescription and reimbursement, and as antifibrotics have only been proven effective in RA-ILD from 2019 onwards, this might have contributed to a lower prevalence in the ICD subgroup [15–17]. The high residual heterogeneity (*I*^2^ = 94.98%) underscores that other sources of heterogeneity, such as study design and variation in diagnostic criteria, likely also play a major role.

### Challenges in diagnosis and definitions

An important challenge in ILDs and RA is the plethora of definitions used in clinical research. Classification criteria are there to guide rheumatologists in their diagnostic process but are not unifying, diagnostic or research criteria [1, 18, 19]. Additionally, even though a specific diagnostic tool might have been used, cut-offs varied and definitions used to define radiographic findings differed. Many studies deviated from the gold standard in ILD diagnosis, namely the multidisciplinary team discussion (MDTD). This heterogeneity in methods and diagnosis undoubtedly leads to a heterogeneity in results. The recent arrival of the American Thoracic Society interstitial lung abnormalities guidelines provides a better clinical and research framework to diagnose interstitial lung abnormalities and ILD in the setting of RA and promises a positive impact on future research [20].

### Strengths and limitations

A major strength of this meta-analysis is the use of a very comprehensive search method with broad inclusion criteria as well as the formal bias evaluation. However, this meta-analysis is limited because of the very substantial heterogeneity and the potential variation in diagnostic thresholds in the included studies. As an inherent risk in a meta-analysis of publications with high heterogeneity [21], it is therefore not possible to provide a clear, statistically sound answer regarding the exact prevalence of RA-ILD. However, this study highlights the critical need for new standardized and methodologically robust epidemiological research to further explore the true prevalence of ILD in patients with RA and to set the scene for screening protocols. In addition, although we used the AXIS tool to assess reporting quality, AXIS mainly evaluates reporting clarity and study description and does not capture key determinants of ILD prevalence (e.g. diagnostic sensitivity, RA duration, smoking, comorbidity burden or systematic imaging) nor population bias. Furthermore, treating AXIS as a continuous score may not reflect the true magnitude of bias.

Missing data represented an additional limitation. Several studies did not clearly specify whether CT imaging was performed using a dedicated high-resolution protocol or conventional CT, precluding reliable quantification of HRCT use. Given the higher sensitivity of HRCT for detecting early and subclinical ILD, this may have influenced prevalence estimates. Moreover, information on the presence or absence of respiratory symptoms was frequently not reported, limiting our ability to distinguish between clinically manifested and subclinical disease.

Additionally, formal adjustment for diagnostic sensitivity was not feasible, as sensitivity and specificity data were inconsistently reported across studies, precluding such modelling. This likely contributed to the substantial residual heterogeneity observed and should be considered when interpreting the pooled prevalence estimates.

A further limitation of this review is that RA treatment and exposure to environmental pollution were not assessed in the meta-regression analysis due to the incomplete availability of data in the included studies. This could have influenced our findings, as RA treatment has a significant impact on the prevalence of ILD, as methotrexate, abatacept, rituximab and tocilizumab have shown promising results in the control of RA-ILD [100–104]. Furthermore, pollution, especially particulate matter with a diameter ≤2.5 μm plays an important role in the risk for development of RA-ILD [105]. Moreover, treatment practices and pollution exposure vary substantially across countries, between cities within the same country, across healthcare systems and over time, all of which are strongly associated with ILD risk, disease severity and likelihood of detection. Together, these factors likely contribute, at least in part, to the residual heterogeneity observed in the meta-regression model.

### Clinical implications

Since the 1960s, the World Health Organization has provided us with 10 key principles for setting up screening programs [22]. The first condition they proposed is that ‘the condition sought should be an important health problem’. RA-ILD is known to be associated with high mortality, and its clinical impact has gained increasing attention in recent years [23]. Beyond its contribution to morbidity and mortality, understanding disease prevalence is fundamental to assessing the significance of a comorbidity. With the recent arrival of guidelines from the ACR and American College of Chest Physicians and those of the European Respiratory Society and European Alliance of Associations for Rheumatology [106], screening will become an even more important part of clinical practice [24]. Therefore, studies like this are essential not only to estimate prevalence but also to evaluate the quality of evidence on which those estimates and screening programs are based.

## Conclusion

Due to the extreme heterogeneity and evidence of small study effects, this meta-analysis cannot provide a conclusive prevalence rate for RA-ILD. However, it clearly demonstrates that prevalence is highly dependent on the diagnostic modality used. Prevalence estimates derived from low-bias and multimodal diagnostic studies (≈10–13%) likely provide a more clinically meaningful approximation than the overall pooled estimate. Nevertheless, even these figures should be interpreted with caution, as residual heterogeneity and potential selection bias may still contribute to overestimation. These estimates are therefore best regarded as informed approximations rather than definitive prevalence rates.

## Supplementary Material

rkag045_Supplementary_Data

## Data Availability

The data underlying this article will be shared upon reasonable request to the corresponding author.
