# Supplementary material for: The prevalence of interstitial lung disease in rheumatoid arthritis: a systematic review
Source: Rheumatol Adv Pract. 2026 Apr 11;10(2):rkag045. doi: 10.1093/rap/rkag045 (PMC13130208; doi:10.1093/rap/rkag045)
Supplement: rkag045_Supplementary_Data [file rkag045_supplementary_data.zip › 25-264 Supplementary Material.docx]

## Supplementary data

### Supplementary File S1: Search Strategy

#### 1. Embase (conference abstracts excluded):

#1) 'interstitial lung disease'/exp OR ‘interstitial lung disease*’:ti,ab,kw OR ‘interstitial pneumopath*’:ti,ab,kw OR ‘diffuse parenchymal lung disease*’:ti,ab,kw OR ‘diffuse parenchymal pulmonary disease*’:ti,ab,kw OR ‘diffuse parenchymal pulmonary disorder*’:ti,ab,kw OR ‘interstitial lung disorder*’:ti,ab,kw OR ‘interstitial pulmonary disease*’:ti,ab,kw OR ‘interstitial pulmonary disorder*’:ti,ab,kw OR ‘ILD’:ti,ab,kw OR ‘interstitial pneumonia*’:ti,ab,kw OR ‘interstitial pneumonit*’:ti,ab,kw OR ‘Pulmonary Fibros*’:ti,ab,kw NOT 'conference abstract'/it

#2) 'rheumatoid arthritis'/exp OR ‘rheumatoid arthritis’:ti,ab,kw OR ‘RA’:ti,ab,kw OR ‘arthritis deformans’:ti,ab,kw OR ‘arthrosis deformans’:ti,ab,kw OR ‘beauvais disease’:ti,ab,kw OR ‘chronic articular rheumatism’:ti,ab,kw OR ‘chronic polyarthritis’:ti,ab,kw OR ‘chronic rheumatoid arthritis’:ti,ab,kw OR ‘inflammatory arthritis’:ti,ab,kw OR ‘primary chronic polyarthritis’:ti,ab,kw OR ‘rheumarthritis’:ti,ab,kw OR ‘rheumatic arthritis’:ti,ab,kw OR ‘rheumatic polyarthritis’:ti,ab,kw NOT 'conference abstract'/it

#1 AND #2 => 5.613 hits

#### 2. Pubmed (including MEDLINE):

#1) "Lung Diseases, Interstitial"[Mesh] OR “interstitial lung disease*”[tiab] OR “ILD”[tiab] OR “Diffuse Parenchymal Lung Disease*” [tiab] OR “Interstitial Pneumonia*”[tiab] OR “Interstitial Pneumonit*”[tiab] OR “Pulmonary Fibros*”[tiab] OR “Diffuse parenchymal pulmonary disease*”[tiab] OR “Diffuse parenchymal pulmonary disorder*”[tiab] OR “Interstitial lung disorder*”[tiab] OR “Interstitial pneumopath*”[tiab] OR “Interstitial pulmonary disease*”[tiab] OR “Interstitial pulmonary disorder*”[tiab]

#2) "Arthritis, Rheumatoid"[Mesh] OR “Rheumatoid arthritis”[tiab] OR “RA”[tiab] OR “arthritis deformans”[tiab] OR “arthrosis deformans”[tiab] OR “beauvais disease”[tiab] OR “chronic articular rheumatism”[tiab] OR “chronic polyarthritis”[tiab] OR “chronic rheumatoid arthritis”[tiab] OR “inflammatory arthritis”[tiab] OR “primary chronic polyarthritis”[tiab] OR “rheumarthritis”[tiab] OR “rheumatic arthritis”[tiab] OR “rheumatic polyarthritis”[tiab]

#1 AND #2 => 3.323 hits

#### 3. CENTRAL (Cochrane library) and Cochrane Database of Systematic Reviews (Cochrane library):

#1) [mh "Lung Diseases, Interstitial"]

#2) ((“interstitial lung” NEXT disease*) OR “ILD” OR (“Diffuse Parenchymal Lung” NEXT Disease*) OR (Interstitial NEXT Pneumonia*) OR (Interstitial NEXT Pneumonit*) OR (Pulmonary NEXT Fibros*) OR (“Diffuse parenchymal pulmonary” NEXT disease*) OR (“Diffuse parenchymal pulmonary” NEXT disorder*) OR (“Interstitial lung” NEXT disorder*) OR (Interstitial NEXT pneumopath*) OR (“Interstitial pulmonary” NEXT disease*) OR (“Interstitial pulmonary” NEXT disorder*)):ti,ab,kw

#3) [mh "Arthritis, Rheumatoid"]

#4) (“Rheumatoid arthritis” OR “RA” OR “arthritis deformans” OR “arthrosis deformans” OR “beauvais disease” OR “chronic articular rheumatism” OR “chronic polyarthritis” OR “chronic rheumatoid arthritis” OR “inflammatory arthritis” OR “primary chronic polyarthritis” OR rheumarthritis OR “rheumatic arthritis” OR “rheumatic polyarthritis”):ti,ab,kw

#5) #1 OR #2

#6) #3 OR #4

#7) #5 AND #6 => 133 hits

Cochrane Central Register of Controlled Trials: 132 hits

Cochrane Database of Systematic Reviews: 1 hit

#### 4. Web of Science Core Collection:

Selected on:

Science Citation Index Expanded

(SCI-EXPANDED)--1955-present

Social Sciences Citation Index

(SSCI)--1956-present

Arts & Humanities Citation Index

(AHCI)--1975-present

Conference Proceedings Citation Index – Science

(CPCI-S)--1990-present

Conference Proceedings Citation Index – Social Science & Humanities

(CPCI-SSH)--1990-present

#1) TS=(“interstitial lung disease*” OR “ILD” OR “Diffuse Parenchymal Lung Disease*” OR “Interstitial Pneumonia*” OR “Interstitial Pneumonit*” OR “Pulmonary Fibros*” OR “Diffuse parenchymal pulmonary disease*” OR “Diffuse parenchymal pulmonary disorder*” OR “Interstitial lung disorder*” OR “Interstitial pneumopath*” OR “Interstitial pulmonary disease*” OR “Interstitial pulmonary disorder*”)

#2) TS=(“Rheumatoid arthritis” OR “RA” OR “arthritis deformans” OR “arthrosis deformans” OR “beauvais disease” OR “chronic articular rheumatism” OR “chronic polyarthritis” OR “chronic rheumatoid arthritis” OR “inflammatory arthritis” OR “primary chronic polyarthritis” OR “rheumarthritis” OR “rheumatic arthritis” OR “rheumatic polyarthritis”)

#1 AND #2 => 2.510 hits

### Supplementary File S2: Forest plot for the low-bias articles between 2013-2022


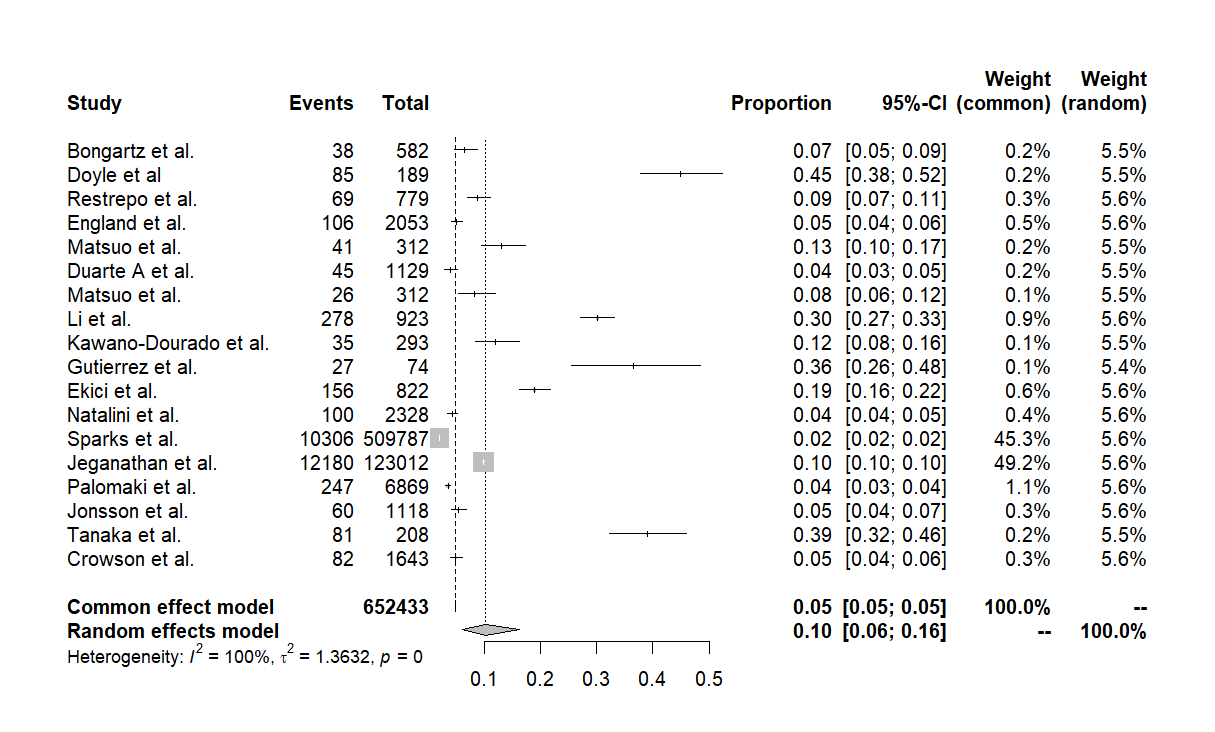


**Supplementary File S2.** Forest plot illustrating the prevalence of interstitial lung disease in patients with rheumatoid arthritis

**Alt text:**

Forest plot showing prevalence estimates of interstitial lung disease in patients with rheumatoid arthritis from studies published between 2013 and 2022 that were assessed as having low risk of bias. Individual studies are presented with their prevalence estimates and confidence intervals, along with a pooled prevalence estimate.
